# Oxaliplatin-Based Intra-Arterial Chemotherapy in Colo-Rectal Cancer Liver Metastases: A Review from Pharmacology to Clinical Application

**DOI:** 10.3390/cancers11020141

**Published:** 2019-01-24

**Authors:** Girolamo Ranieri, Mariarita Laforgia, Patrizia Nardulli, Simona Ferraiuolo, Pasquale Molinari, Ilaria Marech, Cosmo Damiano Gadaleta

**Affiliations:** 1 Interventional and Medical Oncology Unit, National Cancer Research Centre, IRCCS Istituto Tumori Giovanni Paolo II, 70124 Bari, Italy; p.molinari@oncologico.bari.it (P.M.); ilariamare@tin.it (I.M.); c.gadaleta@oncologico.bari.it (C.D.G.); 2 Pharmacy Unit, IRCCS Istituto Tumori Giovanni Paolo II, 70124 Bari, Italy; m.laforgia@oncologico.bari.it (M.L.); p.nardulli@oncologico.bari.it (P.N.); s.ferraiuolo@oncologico.bari.it (S.F.)

**Keywords:** platinum complexes, intra-arterial chemotherapy, arterial catheter, liver metastases

## Abstract

Liver metastases (LM) are often consequences of colo-rectal cancer (CRC)and the majority of patients have unresectable LM. Oxaliplatin-based intravenous chemotherapy represents the gold standard treatment for CRC. Intravenous oxaliplatin has several side effects i.e., nephrologic, hematologic and neurological toxicity. Moreover, hepatic arterial infusion (HAI) of antitumor drugs deeply modifies the treatment of LMCRC due to the knowledge that LM are perfused by the hepatic artery network, whereas healthy tissue is perfused by the portal vein. Therefore, oxaliplatin-based HAI becomes an interesting possibility to treat LMCRC. The aim of this review is to shed light on the important impact of the oxaliplatin-based chemotherapy from a non-conventional clinical point of view, considering that, being universally accepted its antitumor effect if administered intravenously, fragmentary information are known about its clinical applications and benefits deriving from intra-arterial administration in loco-regional chemotherapy.

## 1. Introduction

Platinum-based chemotherapy still represents the gold standard treatment for different solid tumors, though its history dates back to 1965, when Rosenberg casually discovered the antimitotic activity of a Platinum(II) complex delivered in a saline solution on the growth of bacteria in a chamber equipped with a set of platinum electrodes [1].In this environment, bacteria changed in their morphology, generating very long filaments; however, this effect was not due to the electric field directly, but to the electrolysis products produced by the platinum electrodes, among which the most represented was ammonium chloroplatinate [NH_4_]_2_[PtCl_6_]. Further experiments revealed that the active complex on bacteria was a neutral species derived from ammonium chloroplatinate after ultraviolet (UV) light exposure. This substance existed in two isomeric forms, cis and trans, but only the former showed to be an active compound.

**Figure 1:**
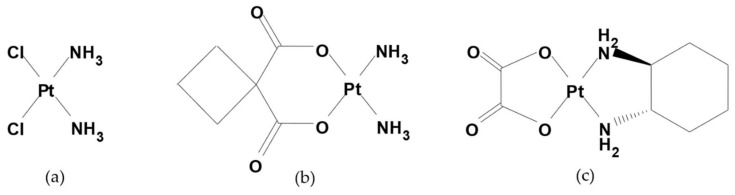
Chemical structure of (**a**) Cisplatin, (**b**) Carboplatin and (**c**) Oxaliplatin.

Hence, the scientific research and development of new platinum complexes has never stopped. Cisplatin or cis-diaminodichloroplatinum (II) complex, is the starter of this peculiar class of antitumor drugs which, through a cycle-unspecific mechanism, act by coordinating the N7 guanine of DNA just through the platinum atom. This link generates a bending of the DNA double helix and, consequently, the processes of replication are inhibited and the programmed mechanism of cellular death, so called apoptosis, is activated. By substituting the two chlorine atoms and complicating the two aminic groups and the steric burden around the coordination plane of the metal atom, it is possible to synthesize in a laboratory a certain number of cisplatin derivatives (carboplatin was the first one [2]) which maintain their antitumor activity and, in some cases, have a more stressed tropism for specific tissues [3], such as oxaliplatin, the platinum(II) complex having the oxalic acid and the (*R,R*)-cyclohexandiamine as ligands for the metal atom (Figure 1), which is largely used in clinical oncology for different solid tumors such as colo-rectal, stomach, pancreatic and ovarian cancer, but also in oncohaematologic diseases as in the GIfOx/R-GIfOx chemotherapy schedule via an intravenous (IV) route.

Oxaliplatin, with respect to cisplatin and carboplatin, has a minor nephrologic and hematologic toxicity, respectively, while its most frequent acute side effect is a reversible peripheral neuropathy, such as paresthesia and dysesthesia completely resolving within 6–8 months after the end of therapy, depending on the cumulative dose administered [4].

In colo-rectal cancer (CRC), oxaliplatin plays an important role via IV route in combination with folinic acid and 5-Fluorouracile/capecitabine.

CRC represents a public health burden all over the world, being the third most frequent tumor disease with its 945,000 patients and the third cause of death with its 610,000 victims a year [5].

Liver metastases (LM) are often consequences of CRC, in fact it is estimated that at the diagnosis of CRC, 15–25% of patients have LM and another 50% will develop LM at a certain point of the disease [6]. Surgical resection of LM generally leads to long-term survival [7,8,9,10], but a large number of the clinical cases have not resectable disease and systemic chemotherapy is still the only authorized standard treatment. The most common regimens in metastatic colo-rectal cancer as first line chemotherapy include the association FOLFOX with monoclonal antibodies on the basis of the histological and molecular characterization either of the primary tumor or of its metastases. Bevacizumab and cetuximab/panitumumab are the principal monoclonal antibodies in the first line of therapy and the oncologist’s choice principally depends on the expression of mutant or wild type KRAS, respectively. In the second line of therapy, the anti-VEGF aflibercept in association with FOLFIRI regimen is a relatively recent treatment after failure with the oxaliplatin-based chemotherapy regimen. Monoclonal antibodies, deriving from immunoglobulins, are well known first examples of immunotherapy, even if nowadays this term is always associated to check-point inhibitors such as nivolumab, which in July 2017, rapidly received authorization by the US FDA for the treatment of metastatic colo-rectal cancer with DNA mismatch repair-deficient (dMMR)/microsatelliteinstability-high (MSI-H) colo-rectal cancer (associated to a poor prognosis), after the failure of conventional chemotherapy [11].

In these relatively limited therapeutic options, locoregional hepatic intra-arterial (HAI) administration becomes a possible way to treat LMCRC [12], granting more broad ways of treatment.

The aim of this review is to shed light on the important impact of the oxaliplatin-based chemotherapy from a non-conventional clinical point of view, considering that, being universally accepted its antitumor effect if administered intravenously, fragmentary information are known about its clinical applications and benefits deriving from intra-arterial administration in loco-regional chemotherapy.

## 2. Chemotherapy in Hepatic Arterial Infusion (HAI)

Liver Metastases in Colo-Rectal Cancer (LMCRC) is the most frequent complication of the primary tumor, with 15–25% of patients having it at the time of diagnosis and up to 60% of patients developing it in the course of the disease. Surgical resection of LM is generally associated with a long-term disease-free survival, but unfortunately only 10% to 20% of patients are eligible for surgery or suitable to other local treatments, such as radiofrequency ablation, that could induce a cure or response. The short-term prognosis of inoperable patients has started the exploration of new or alternative clinical practices aiming at the conversion to resectability for unresectable LMCRC [13].

**Figure 2:**
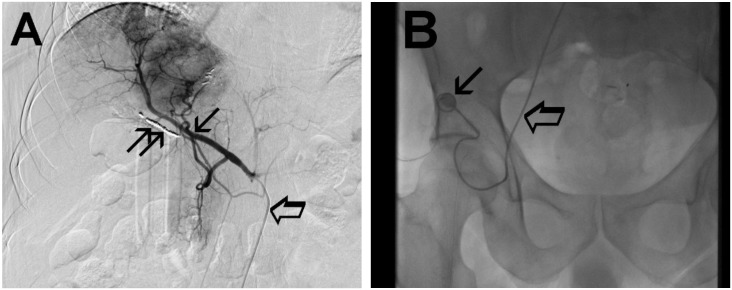
The arterial phase of the contrast angiogram shows the subcutaneous port and the hepatic-artery catheter (**A**.) The arterial phase of the contrast angiogram shows the hepatic-artery (thin arrow), the preparation of the vascular bed (double thin arrow) and the hepatic-artery catheter (large arrow). (**B**.) The arterial phase of the contrast angiogram shows the subcutaneous port (thin arrow) with its connection system and the hepatic-artery catheter (large arrow).

Hepatic Arterial Infusion (HAI) of antitumor drugs deeply modified the treatment of LMCRC and is based on the knowledge that LM are perfused by the hepatic artery network, whereas healthy tissue is perfused by the portal vein [14,15]. The arterial catheter for HAI is implanted after contrast angiogram (Figure 2) as described by Kemeny and Fata and connected to the subcutaneous placed port in the right inguinal position [16].

In this regard, it is important to underscore that in the first studies, HAI was administered by means of an external wearable infusion pump and it was complicated by higher rates of catheter dislodgement, catheter sepsis and irregular chemotherapy flow perfusion, so that subsequently, a total implantable pump was developed and this device was associated with a reduction rate of the above complications [17].

Up to now, HAI can be delivered by means of either an implanted pump or a port surgically implanted in a subcutaneous pocket on the abdominal wall and in the right inguinal fossa, respectively, and both pump and port are connected to a catheter placed during laparotomy into the gastroduodenal; in this manner, the administered drug can be distributed into the hepatic arterial blood [18].

If a concurrent oncological major surgery is not required, the interventional radiology percutaneous placement of the port catheter device by axillary or femoral access is preferred in our institution [19], above all because radiologic positioning of the catheter-port device is a minimally invasive safe technique to perform [20].

After the above access, the catheter is introduced into the celiac axis and then into the hepatic artery with the side hole of the catheter about 10 cm from its distal tip (Figure 2A). This procedure is performed under an angiographic guide and is completed with the embolization of the gastroduodenal artery, right gastric artery and any other branches of a proper hepatic artery with a metallic helix to avoid toxicity due to extrahepatic perfusion [21]. Finally, the catheter is heparinized and connected with the port-a-cath chamber allocated in a pocket in the right inguinal area (Figure 2B).

The common complications due to the catheter implantation are arterial and catheter thrombosis, extrahepatic perfusion, catheter tip migration, catheter infection, and hemorrhage of the task. The complications were initially reported to vary between 30 and 79%, but currently are reduced to 20% and are mostly minor and salvageable [22].

With special regard to the chemotherapeutic agent employed for HAI, due to their pharmacokinetic properties, floxuridine and 5-fluorouracil have been the classical most commonly used drug. More recently, oxaliplatin has also been employed. Up to now, a lot of clinical trials have demonstrated positive results of HAI in the therapy of only liver colo-rectal cancer metastases [23].

Although the technique HAI for the treatment of liver metastases from colo-rectal cancer has been developed over more than 40 years, it is not routinely performed in clinical practice due to several issues: first of all, published studies have been sometimes heterogeneous, with different regimens of intra-arterial drugs, associated or not with systemic chemotherapy; the second issue is the particular technical expertise required for catheter placement and management; finally, the lack of education and training in HAI therapy for interdisciplinary medical professionals such as medical oncologists, interventional radiology and surgeons. Although HAI is now adopted in a few specialized institutions, consensus statements indicating the utilization of HAI in LMCRC therapy have been published in the last few years, increasing the scientific interest to the clinical application of this therapy.

In 2014, an expert group discussed the role of HAI in the contemporary management of patients with LMCRC. Using a consensus process, the experts developed some statements [24]:

HAI chemotherapy should be given in combination with systemic chemotherapy.

HAI chemotherapy should be offered in the context of a multidisciplinary program that includes expertise in hepatobiliary surgery, medical oncology, interventional radiology, nursing, and nuclear medicine (and we add pharmacists).

HAI chemotherapy in combination with systemic therapy should be considered in patients with unresectable LMCRC who have progressed on first-line systemic treatment. In addition, HAI chemotherapy is acceptable as first-line treatment in patients with unresectable LMCRC.

HAI chemotherapy is not recommended in the setting of extrahepatic disease outside the context of a clinical trial.

HAI chemotherapy in combination with systemic therapy is an option for selected patients with resected LMCRC.

These consensus statements provide a framework that clinicians who treat patients with LMCRC can use when considering treatment with HAI.

## 3. Chemotherapic Drugs in HAI

Floxuridine (FUDR) was the most widely administered drug through HAI, because of its high hepatic extraction rate of 95%, its short half-life and its tumor exposure almost 400 times greater than systemic administration [25,26]. In particular, its high hepatic extraction rate has been the reason for its large application in intra-arterial chemotherapy for years, also thanks to the obviously limited systemic toxicity, particularly in combination with systemic antitumor agents.

In previously reported phase I and II studies, and then with the clinical use according to ministerial statements and therapeutic indications of HAI FUDR, also in combination with systemic chemotherapies, they showed a response rate (RR) between 52 and 75% in non-naïve patients and even higher in chemotherapy naïve ones. Also significant in the reported trials is the conversion to resection in 47% of a heavily pretreated cohort of patients [27,28].

FUDR belongs to the antimetabolite group of anticancer drugs such as 5-Fluorouracile (see below), and its common medium dose through HAI administration is about 0.12 mg/kg/die via continuous infusion for 14 days.

HAI 5-FU is nowadays an affirmed procedure for the treatment of LM, principally deriving from breast cancer [19]. Notwithstanding this, clinical results for HAI 5-FU for LMCRC are similar to FUDR ones at a medium dose of 1000 mg/m^2^ over 44 hours by continuous infusion.

Only one phase II clinical trial is present in literature with HAI cisplatin in LMCRC, compared to its IV administration [29]. The trial evidenced the similarity of HAI cisplatin and HAI FUDR in terms of response to treatment, being ORR 52% both in HAI and in IV administration, but with a lower toxicity in the HAI arm. No further studies or comparisons of cisplatin with other drugs are known at the moment.

Other recent and less recent studies tested old antitumor drugs through an intra-arterial route, such as irinotecan [30] combined to beads as salvage therapy or mitomycin C [31] in the form of hypoxic perfusion in multifocal liver metastases.

## 4. Oxaliplatin Chemistry and Pharmaceutical Properties

With respect to cisplatin, oxaliplatin presents the (*R,R*)-cyclohexandiamine ring instead of the ammonia groups and the oxalic acid instead of the two chlorine atoms, as ligands for platinum. If nitrogen is a strong ligand for the metal, the oxygen atoms on the other half of the coordination plane belonging to the dicarboxylic groups of oxalic acid are weak ligands for platinum, so that a saline solution containing choride ions (as in NaCl 0.9%), which are able nucleophiles towards the metal, destroy the molecule, provoking the detachment of the dicarboxylic group and the deactivation of the drug before administration. As a consequence, galenic products of oxaliplatin must be prepared in glucose 5% solution which preserves the molecule from chemical instability independently from the final concentration [32].

Being a cytotoxic drug [33], considered by IARC (International Agency for Researh on Cancer) as a potential carcinogenic substance [34], oxaliplatin must be handled in controlled conditions in a negative pressure laboratory endowed with vertical laminar flow cabins in order to preserve both the galenic product and the operators’ health and environment from possible contaminations; for this reason, operators who handle it must be properly instructed.

When prepared for intrahepatic infusion, the drug is diluted in the minimum volume of 100 mL of glucose 5% solution within an appropriate reservoir connected to an electronic pump, set up by an operator that selects the flow in mL/min and the duration of the infusion, according to the therapeutic and clinical needs. The reservoir does not deliver the drug until its connection to an intra-arterial catheter previously inserted through a surgical procedure on the hepatic artery and the activation of the pump.

Oxaliplatin diluted solutions in glucose 5% are chemically and physically stable also at relatively high concentrations up to 1.3 mg/mL, because its chemical structure remains unmodified and there are no aggregates of drug precipitate for a relatively long time [35,36].

## 5. Oxaliplatin Pharmacology and Pharmacokinetics

After IV administration, through a slow infusion of about 2 hours at a maximum dose of 130 mg/m^2^, oxaliplatin enters the cellular membranes (through still partially unclear mechanisms) and in the cytoplasm the oxalic moiety detaches from the metal in favor of water molecules; the aquospecies leave the cytoplasm to reach the nucleus, where the platinum atom coordinates the N7 of a guanine base. This event modifies the distance between the nucleic bases G–C within the DNA double helix, causing its kinking and, at the end, cell death [37].

The pharmacokinetic differences among platinum drugs are generally attributed to the structure of their leaving groups, in which particular less easily displaced leaving groups exhibit reduced plasma protein binding, longer plasma half-lives, and higher rates of renal clearance [38,39,40].

A preclinical report on the pharmacokinetic advantage of HAI oxaliplatin administration with respect to IV route and to cisplatin in rabbit VX2 tumor model (an epidermoid tumor induced by the Shope papilloma virus which well represents liver cancer) was conducted in 2004 [41], which measured pharmacokinetic parameters by atomic absorption spectrometry and revealed that there was no difference in bioavailability between HAI oxaliplatin and IV cisplatin, while significant differences in various tissue concentrations were reported between HAI and IV oxaliplatin, in favor of the former.

In humans, after oxaliplatin IV infusion, platinum accumulates into three compartments: plasma-bound platinum, ultrafilterable platinum and platinum associated with erythrocytes.

Approximately 85% of the total platinum is bound to plasma proteins at 2 to 5 hours post infusion [42], and plasma elimination of total platinum and ultrafiltrates is biphasic, being the T_1/2_ in the first phase and T_1/2_ in the terminal phase 26 minutes and 38.7 hours, respectively, for total platinum and 21 minutes and 24.2 hours, respectively, for ultrafilterable platinum [42,43]. Similarly to cisplatin, a prolonged retention of oxaliplatin is observed in red blood cells, but unlike cisplatin it does not accumulate to any significant level after multiple courses of treatment [42,43]. Oxaliplatin is eliminated predominantly by the kidneys, with more than 50% being excreted in urine at 48 hours.

As far as HAI oxaliplatin administration is concerned, the first strong phase I study on its pharmacokinetics was reported by Kern in 2001 [43] with escalating doses of HAI oxaliplatin aiming at determining its highest tolerated dose and collecting information about its metabolism and first pharmacokinetic data by using flameless atomic absorption spectrometry on the plasma level after ultrafiltration and on renal excretion to determine platinum concentration. The study demonstrated that the oxaliplatin pharmacokinetic profile administered by HAI highly differed from the IV route for terminal halflife (17.8 ± 9.3 hours vs. 27.3 ± 10.6 hours, respectively) and AUC (17.76 ± 7.8 mcg per h/mL vs. 20.17 ± 6.97 mcg per h/mL), but is similar in renal clearance (135 ± 55 mL/min vs. 121 ± 56 mL/min) and renal elimination (49% ± 14% vs. 54% ± 20%). This discovery opened the door to the assumption of the reduced systemic availability of the drug by HAI associated to less toxicity and augmented availability to the target organ.

The second and stronger study on oxaliplatin pharmacokinetics on man was reported by Guthoff in 2003, when for the first time a value of its liver extraction rate was given [44], thanks to a previous in-vitro experiment by a researcher of the same scientific group, who used fresh tumor cells isolated from LMCRC mimicking HAI conditions in the human tumor colony forming assay [45]. The resulted cytotoxicity on the selected cell lines was particularly exciting and was the starting point for the developing of a phase II clinical trial specifically built, involving patients with isolated unresectable LMCRC, for which oxaliplatin pharmacokinetics was followed through peripheral venous blood collected before, during and after arterial infusion and quantified by liquid chromatography with post-column derivatization [44]. Comparing the AUC values after IV administration (161 ± 23 mcg per min/mL) with the HAI data (85.3 ± 13.7 mcg per min/mL) for the same administered dose (85 mg/m^2^), it was possible to affirm that oxaliplatin had a liver extraction ratio of 0.47, that means that only approximately half of the intra-arterially administered oxaliplatin reaches the general circulation, which is the reason of the very favorable safety profile of this old drug in new and innovative procedures.

## 6. Toxicity in HAI Chemotherapy

One of the most significant side effects while administering through hepatic arterial blood is the modification of liver enzyme levels (alkaline phosphatase, lactate dehydrogenase and transaminases) and bilirubin that can be responsible for the interruption of the regular course of the therapy. Nevertheless, the most dangerous side effect is undoubtedly the dilation of hepatic sinusoidal capillaries and the atrophy of hepatic cells, a progressive degenerative process which can lead to cirrhosis and hepatic necrosis [46].

For this reason and also for its limited, almost unique, clinical use in HAI, about 10 years ago, FUDR disappeared from the Italian pharmaceutical market in favor of a more versatile drug, 5-Fluorouracile, another antimetabolite antitumor drug authorized both for intra-arterial and IV administration, as reported in its technical sheet.

Also 5-FU side effects are comparable to FUDR and its clinical use gave light to an important side effect that is often responsible for dose reductions or alternative flows or chronomodulation of infusion, that is, patients refer a stomachache and heartburn that seems to be connected to inflammation of hepatic arterial endothelium, overcoming the six/seven doses. Hence, the necessity for a functional pre- and post intra-arterial medication with desamethazone. The management of these side effects of FUDR and 5-FU is not simple, but the importance to exploit the great benefits of locoregional chemotherapy has always been deeply felt.

The most important side effect of oxaliplatin via IV route is neurotoxicity, which develops in the two following stages. First, a tingling of the extremities occurs and usually resolves within a few days, but with repeated dosing, symptoms may last longer between following administrations but are generally are short in time and not cumulative. Laryngopharyngeal spasm and cold dysesthesias also have been reported but are not associated with significant respiratory symptoms; they can be prevented by prolonging the duration of infusion. A second stage of neuropathy, similarly to cisplatin, affects the extremities and increases with repeated doses. One of the first phase I studies by Extra et al., thanks to the help of electromyography, revealed axonal sensory neuropathy, but unmodified nerve conduction velocities [38]. Peripheral nerve biopsies performed in this study showed decreased myelinization and replacement with collagen pockets; in this stage, the neurologic effects of oxaliplatin appear to be cumulative, more pronounced and of greater duration with successive cycles, but unlike cisplatin, they are reversible with the end of treatment. In a cohort of patients treated, Sugihara et al. reported that for the majority of patients who experienced grade 2 or higher neurotoxicity, the regression of their symptoms occurred within 4 to 6 months [47]. Neither ototoxicity nor nephrotoxicity is observed and myelosuppression appears above all in association with regimens of oxaliplatin, rather than as a single agent, while nausea and vomiting generally respond to 5-HT 3 antagonists.

At the moment, no reported important side effects are known for oxaliplatin administered via a hepatic intra-arterial route, so that in the near future, this therapeutic choice could become a form of long-lasting maintenance therapy in patients with inevitable progressing disease, as deducted by the toxicity data reported in the following clinical trials.

IV and intra-arterial oxaliplatin toxicities are summarized in Table 1, by using the frequency of occurrence in the patients of the examined clinical trials and also taking into account the data of oxaliplatin technical sheet for intravenous administration.

All adverse events while administering oxaliplatin are more common via intravenous rather than via intra-arterial route, except for thrombosis whose frequency is the same for both, but with a substantial difference in the breaking cause, being intrinsic in the insertion of the arterial catheter. It presumably plays a more important role with respect to the direct involvement of the drug, although in a more concentrated solution, as in IV administration [48].

## 7. Clinical Trials with HAI Oxaliplatin

Unfortunately, the clinical options to treat LMCRC are really few; for this reason, the re-discovery of a well-known and well-tolerated drug such as oxaliplatin, in the first steps of LMCRC, without extrahepatic lesions, represents a key turning point in the management of this clinical condition that often rapidly degenerates.

Kern et al. in a phase I study combined HAI oxaliplatin (25 mg/m^2^ with dose increments of 25 mg/m^2^ every 3 weeks) with IV 5-Fluorouracil (5-FU) and leucovorin (LV) according to “de Gramont” regimen in patients with LMCRC [43]. The limiting toxicities observed at an oxaliplatin dose of 150 mg/m^2^ were leukopenia, occlusion of the hepatic artery and acute pancreatitis, so thatthe recommended dose was 125 mg/m^2^ every 3 weeks with an objective RR (ORR) of 59%.

Mancuso et al. in a phase I trial evaluated the feasibility, the dose-limiting toxicity (DLT) and toxicity profile of HAI oxaliplatin in pretreated patients with LMCRC [49]. Toxicity grade 1–2 was haematological (mainly leukopenia), asthenia, mucositis, neurotoxicity and abdominal pain, whilesevere complications were gastrointestinal (41%, main DLT) i.e., peptic ulcer and gastritis andsecondary complications were arterial thrombosis and pump pocket haematoma. The ORR and the median OS were 67% and 19 months, respectively.

Fiorentini et al. analyzed the antitumor efficacy of HAI oxaliplatin in previously treated patients with unresectable LMCRC [50]. As far as the response to therapy is concerned, they reported 33% of partial response and 17% of stabilization of disease, while the median time to failure was 14 weeks and the median overall survival (OS) was 13 months. Toxicity was mild, mainly consisted of gastrointestinal (nausea/vomiting or abdominal pain), haematological (anaemia and thrombocytopenia grade 1–2, leukopenia grade 3 in two cases), sensory neuropathy and obliteration of the hepatic artery (in one case).

Ducreux et al. evaluated in a phase II trial the efficacy of HAI oxaliplatin combined with “de Gramont” regimen in patients with unresectable LMCRC [51]. The ORR was 64% and 18% of patients could proceed to surgical resection of their metastases with curative intent. The median OS and progression-free survival (PFS) were 27 and 27 months, respectively, the most frequent toxicity was gastrointestinal (nausea/vomiting or diarrhea) and the most frequent grade 3–4 toxicity was neutropenia (38%).

Boige et al. analyzed HAI oxaliplatin combined with IV LV (200 mg/m^2^) and bolus 5-FU (400 mg/m^2^) on day 1 followed by infusional 5-FU (2400 mg/m^2^ over 48 h, modified “de Gramont” regimen) [52] in patients who had previously received, and mostly failed, a median of two lines (range 1–5) of systemic chemotherapy (irinotecan and/or oxaliplatin plus 5-FU/LV). The ORR was 55% and tumor response allowed a further R0 surgical resection or radiofrequency ablation of initially unresectable LM in 18% of patients, while the median PFS and OS were 7 months and 16 months, respectively. Complications of HAI, especially extrahepatic perfusion and tip migration or catheter obstruction, were more frequent with percutaneously implanted ports than with surgically implanted ports, while the most frequent adverse events were neuropathy, nausea/vomiting or abdominal pain and neutropenia.

Tsimberidou et al. conducted a phase I study of HAI oxaliplatin combined with systemic 5-FU/ leucovorin and bevacizumab in 57 patients with advanced solid tumors with LM, including 29 LMCRC patients [53] with a median number of prior therapies of four lines (range 1–8). For these last patients, the ORR was 43%, the median PFS and OS were not evaluated, while for the other patients the median OS was 9 months and the median PFS was 3 months. The most common toxicities were thrombocytopenia, fatigue, nausea/vomiting, constipation and diarrhea.

Tsimberidou et al. also analyzed HAI oxaliplatin in combination with IV bevacizumab, with or without HAI or IV fluorouracil and/or leucovorin and/or cetuximab in 76 patients with advanced cancer and predominant LM [54]. In 58 patients with LMCRC, the ORR was 18% and in particular ORR was 6% in patients with mutant or unknown KRAS not given cetuximab and 12% in wild-type KRAS treated with cetuximab combination therapy. The median time to treatment failure was 3 months in patients with mutant KRAS and 4 months in patients with wild-type KRAS and the median OS was 12 months for those with wild-type KRAS and 7 months for those with mutant KRAS. The most common possible treatment-related toxicities were hypertension, nausea, fatigue and high transaminases.

Allard et al. interestingly compared the incidence of complete pathologic response (CPR) and severe oxaliplatin-related lesions (SOxL) between IV and HAI oxaliplatin administration plus IV 5-fluorouracil, leucovorin, bevacizumab or cetuximab in patients with unresectable LMCRC [55]. A CPR was observed significantly more often after HAI (33 vs. 10%) and obviously a CPR markedly prolonged OS and disease-free survival (DFS). The median OS was 114 vs. 42 months in the HAI group and in the IV group, respectively, and the median DFS was 51 vs. 12 months in the HAI group and in the IV group, respectively. However, SOxL occurred more frequently and significantly after HAI administration compared to IV administration, 66 and 20%, respectively, but patients with SOxL did not experience different outcome (median OS of 42 vs. 50 months, respectively).

Volovat et al. evaluated in a phase II study HAI oxaliplatin plus IV 5-fluorouracil, leucovorin and irinotecan (FOLFIRI) in patients with unresectable LMCRC [56]. The ORR was 78% and the median PFS and OS were 20 and 29 months, respectively. The most common grade 1–2 toxicities were gastrointestinal (nausea, vomiting, diarrhea), stomatitis, peripheral neurotoxicity, alopecia and hematological (neutropenia and thrombocytopaenia). Severe toxicities were abdominal pain (70%, grade 3) and neutropenia (21%, grade 3 and 16% grade 4), while a reported complication was catheter obstruction (21%).

Lévi et al. in a European multicenter phase II OPTILIV trial analyzed whether HAI triplet chemotherapy (irinotecan 180 mg/m^2^; oxaliplatin 85 mg/m^2^ and 5-fluorouracil 2800 mg/m^2^) plus IV cetuximab could increase the curative intent resection rate (R0–R1 hepatectomy) to 30% compared to 15% of IV chemotherapy in previously treated patients with wild-type KRAS and unresectable LMCRC [57]. The toxicity of HAI triplet chemotherapy was frequent with grade 3–4 neutropenia (43%), abdominal pain (26%), fatigue (18%) and diarrhea (16%). The ORR was 41%,the median PFS and OS reached 9 and 26 months, respectively. Those patients with R0–R1 hepatectomy had a median OS of 35 months, with 37% alive at 4 years.

Lim et al. analyzed HAI oxaliplatin plus IV 5-fluorouracil, leucovorin, bevacizumab or cetuximab in patients with unresectable LMCRC [58]. Principal toxicity was neurotoxicity (16% of grade 3–4) and neutropenia (22% of grade 3–4). The tumor RR in the first- and second-line was 26.5% and the third- and fourth-line was 11%. The median OS in the first- and second-line was 14 months and in the third- and fourth-line was 8 months; the median PFS in the first- and second-line was 9 months and in the third- and fourth-line was 6 months.

Sato et al. tested in a more recent phase I-II study HAI oxaliplatin plus IV fluorouracil and leucovorin in patients with unresectable LMCRC after systemic chemotherapy failure [59]. In phase I, the recommended dose for oxaliplatin by HAI was estimated in 100 mg/m^2^. In phase II, in patients receiving the recommended dose, the disease control rates for LM were 70%. The 6-month survival rate and the overall survival time were 53% and 7 months, respectively, and there were no adverse reactions equivalent to DLT in any of the patients.

As far as ORR is concerned, Lim et al. showed a lower ORR because they included patients with extrahepatic disease and major hepatic involvement compared to other studies (i.e., Boige et al.) that included only patients without extrahepatic disease or a low rate (less than 30%) of liver involvement. Moreover, in phase II clinical trials, HAI oxaliplatin dose (from 85 to 150 mg/m^2^) and schedule (every 2 or 3 weeks) were variable.

As far as PFS is concerned, the number of lines of chemotherapy previously received by the patients is an important point to consider, i.e., Boige et al. found the worst median PFS, about 7 months. They included patients who received more than two previous lines of chemotherapy compared to Ducreux et al. that included patients who have received none or the first line of IV chemotherapy without oxaliplatin and observed the best PFS to be about 27 months. Another bias that could have influenced PFS was that patients were treated using multiple different systemic chemotherapies (FUFA, FOLFIRI with or without cetuximab or bevacizumab) associated to HAI oxaliplatin.

As far as OS is concerned, Allard et al. compared HAI and IV administration of oxaliplatin in chemotherapy naïve patients, finding the higher median OS (114 months in the HAI group vs. 42 months in the IV group). Recently, in the OPTILIV study, testing a more aggressive regimen, HAI triplet combining 5-FU, irinotecan and oxaliplatin plus IV cetuximab, the median OS was increased to 26 months, confirming the possibility of using an aggressive HAI regimen.

Clinical trials that evaluated HAI oxaliplatin in patients with unresectable LMCRC are summarized in Table 2.

## 8. Conclusions

Since their approval in antitumor therapy some decades ago, platinum complexes have been cornerstones for oncologists in their choice of the best drug for each patient. On the other hand, thanks to their chemical and pharmaceutical versatility, academic and industrial efforts have never stopped in the development of modified platinum molecules to earmark specific research aims and to target therapy [60,61].

The uncommon versatility of oxaliplatin in the treatment of LMCRC has opened a new scenario in the management of often critical diseases also for the lack of alternative therapies or recognized standard treatments by the authorities.

The HAI oxaliplatin by means of an infusion pump connected to the locally inserted catheter represents a less toxic way for the patients to face their localized LM and an innovative and alternative strategy for the physician to approach the multifactor consequences of an uncontrolled disease.

Locoregional chemotherapy, in particular hepatic arterial administration of chemotherapies, has been recognized worldwide as a safe approach in combination with systemic chemotherapy, and the choice of drug to infuse has interested scientists for years. 

With its safety profile, its pharmacokinetic properties and its liver extraction rate of almost 50% with respect to the administered dose, currently, oxaliplatin can be considered the most tolerated drug applied with this interventistic procedure in the treatment of LMCRC.

The studies resumed in this paper shed light on the new hypotheses of work in clinical practice, such as a possible maintenance therapy with HAI oxaliplatin or HAI oxaliplatin in the XELOX regimen after resection, giving HAI adjuvant chemotherapy promising results in different clinical trials [62,63,64,65,66]. The drugs investigated for perioperative HAI after resection are FUDR and 5FU; all the reported studies underline the important improvement of survival in patients who undergo resection followed by adjuvant HAI. To the authors’ knowledge, still no research is public for adjuvant HAI oxaliplatin, but new clinical trials on this topic could bring about surprising clinical results.

## Figures and Tables

**Table 1:** Intravenous and intra-arterial oxaliplatin toxicities.

Toxicity	Intravenous Oxaliplatin	Intra-Arterial Oxaliplatin
Allergic reactions	+++	not known
Anorexia	+++	not known
Neuropathy	+++	++
Anaemia	+++	++
Thrombocytopenia	+++	++
Leukopenia	+++	++
Asthenia	+++	++
Mucositis	+++	+
Alopecia	+++	+
Nausea/Vomiting	+++	++
Diarrhea/Constipation	+++	++
Abdominal pain	+++	++
Hypertransaminasemia	+++	++
Bleeding	+++	+
Thrombosis	++	++
Dyspnea	+++	not known

+++: very common (≥ 1/10); ++: common (≥ 1/100, < 1/10); +: not common (≥ 1/1000 and < 1/100)

**Table 2:** Clinical trials that evaluated HAI oxaliplatin in patients with unresectable liver metastases from colo-rectal cancer.

Reference	Phase of Study	No.of Patients	Previous Treatments	Dose	Systemic Chemotherapy Associated	ORR	mPFS	mOS
Kern et al. 2001 [43]	I	21	Yes or No	25 mg/m^2^ with increments of 25 mg/m^2^	FUFA	59%	n.e.	n.e.
Mancuso et al. 2003 [49]	I	17	Yes	20 mg/m2/day x 5 days every 3 weeks	None	67%	n.e.	19
Fiorentini et al. 2004 [50]	I-II	12	Yes	150 mg/m^2^ every 3 weeks	None or FUFA ±Irinotecan	50%	4 m	13 m
Ducreux et al. 2005 [51]	II	26	None or first line of IV CT without oxal	100 mg/m^2^ every 2 weeks	FUFA	64%	27 m	27 m
Boige et al. 2007 [52]	II	44	Yes (> two lines)	100 mg/m^2^ every 2 weeks	FUFA	55%	7 m	16 m
Tsimberidou et al. 2010 [53]	I	29	Yes (> two lines)	60-175 mg/m^2^ every 2 weeks	FUFA plus bevacizumab	43%	n.e.	n.e.
Tsimberidou et al.2013 [54]	I	58	Yes	140 mg/m^2^ every 3 weeks	FUFA+bevacizumab/cetuximab	12% (KRAS negative) 6% (KRAS positive)	n.e.	12 m (KRAS negative)7 m (KRAS positive)
Allard et al. 2015 [55]	II	68	No	100 mg/m^2^ every 2 weeks	FUFA+cetuximab	n.e.	n.e.	114 m
Volovat et al. 2016 [56]	II	24	Yes or no	85 mg/m^2^ every 2 weeks	FOLFIRI	78%	20 m	29 m
Lévi et al. 2016 [57]	II	64	Yes	85 mg/m^2^ every 2 weeks	HAI irinotecan 180 mg/m^2^+5-FU 2800 mg/m^2^ plus IV cetuximab	41%	9 m	26 m
Lim et al. 2017 [58]	MCS	61	Yes or no	every 2 weeks	FUFA+bevacizumab/cetuximab	11–27%	9–6 m	14–8 m
Sato et al. 2018 [59]	I-II	13	Yes	50-100 mg/m^2^ every 2 weeks	FUFA	70%	n.e.	7 m

FUFA, 5-FU and folinic acid; n.e., not evaluated; m, months; FOLFIRI, 5-fluorouracil, folinic acid, irinotecan.
